# Activated PI3Kδ syndrome – reviewing challenges in diagnosis and treatment

**DOI:** 10.3389/fimmu.2023.1208567

**Published:** 2023-07-20

**Authors:** Sven Vanselow, Volker Wahn, Catharina Schuetz

**Affiliations:** ^1^ Infill Healthcare Communication, Königswinter, Germany; ^2^ Department of Pediatric Respiratory Medicine, Immunology and Critical Care Medicine at Charité University Hospital Berlin, Berlin, Germany; ^3^ Medical Faculty of The Technical University (TU) Dresden, Department of Pediatrics, University Hospital Carl Gustav Carus, Dresden, Germany; ^4^ University Center for Rare Diseases, University Hospital Carl Gustav Carus, Dresden, Germany

**Keywords:** APDS, IEI, inborn errors of immunity, targeted therapy, stem cell transplantation, rare disease, PID, primary immunodeficiency

## Abstract

Activated PI3Kδ syndrome (APDS) is a rare inborn error of immunity (IEI) characterized primarily by frequent infections, lymphoproliferation and autoimmunity. Since its initial description in 2013, APDS has become part of the growing group of nearly 500 IEIs affecting various components of the immune system. The two subtypes of APDS - APDS1 and APDS2 - are caused by variants in the *PIK3CD* and *PIK3R1* genes, respectively. Due to the rarity of the disease and the heterogeneous clinical picture, many patients are not diagnosed until years after symptom onset. Another challenge is the large number of *PIK3CD* and *PIK3R1* variants whose functional significance for developing APDS is inconclusive. Treatment of APDS has so far been mostly symptom-oriented with immunoglobulin replacement therapy, immunosuppressive therapies and antibiotic or antiviral prophylaxes. Additionally, allogeneic stem cell transplantation as well as new targeted therapies are options targeting the root cause that may improve patients’ quality of life and life expectancy. However, the clinical course of the disease is difficult to predict which complicates the choice of appropriate therapies. This review article discusses diagnostic procedures and current and future treatment options, and highlights the difficulties that physicians, patients and their caretakers face in managing this complex disease. This article is based on cohort studies, the German and US guidelines on the management of primary immunodeficiencies as well as on published experience with diagnosis and compiled treatment experience for APDS.

## Introduction

The group of inborn errors of immunity (IEI) currently comprises nearly 500 heritable disorders of immune function and/or regulation (1). While individual IEI disorders are rare, IEIs as a group account for a significant disease and financial burden for healthcare systems (2–4). Current estimates are that around 1 in 2000 people are affected by IEI, with more individuals diagnosed due to increasing awareness amongst healthcare providers (5, 6). The International Union of Immunological Societies (IUIS) classifies IEIs into 10 groups according to the part of the immune system predominantly affected or associated features, e.g. into combined immunodeficiencies (affecting T- and B cell function), predominantly antibody deficiencies (affecting primarily B cell function), immune dysregulatory disorders or others (1).

An important milestone was the introduction of newborn screening for severe T-cell lymphopenia/severe combined immunodeficiencies (SCID). SCID is invariably lethal if left untreated, but can be cured with early hematopoietic stem cell transplantation (7). Some IEIs are included in umbrella terms such as common variable immunodeficiency (CVID), while others have been characterized as distinct entities including X-linked agammaglobulinemia (XLA) (8), chronic granulomatous disease (CGD) (9) or activated PI3Kδ syndrome (APDS) (10, 11). These diseases are not being routinely tested for in newborns, although a few countries include testing for XLA. For many patients, there is a significant delay between symptom onset and diagnosis, oftentimes leading to irreversible organ damage (12).

APDS is a very rare disease. It may be caused by point mutations or deletions in one of the two genes encoding the two phosphoinositide 3-kinase δ (PI3Kδ) subunits. Both gain-of-function (GOF) in *PIK3CD* and loss-of-function (LOF) in *PIK3R1* contribute to the clinical phenotype (13), i.e. the two mechanisms are represented in the pathogenesis of APDS. The disease pattern of APDS has been largely deciphered and may serve as an example to explain how GOF and LOF mutations in a signaling pathway cause disease. The spectrum of symptoms and their severity greatly vary among APDS patients. APDS often manifests early in life with a combination of recurrent infections, lymphoproliferation, and autoimmune disease, and - later in life - with deteriorating lung function or lymphoma. However, many patients may be oligosymptomatic. Indeed, even asymptomatic individuals have been described in literature (14). Consequently, the first hurdle in patient care is diagnosis of affected individuals, which requires attentive general practitioners and efficient diagnostic algorithms. The heterogeneity of the APDS patient collective also poses a challenge for choosing the right therapeutic approach. While symptom-oriented therapy may be appropriate for some patients, others develop life-threatening infections or lymphoma. Accordingly, the consideration of whether to proceed with potentially curative hematopoietic stem cell transplantation (HSCT), associated with potential risks, is not straightforward. APDS patients thus have an unmet need for therapeutic options which address immunodeficiency and lymphoproliferation alike with limited side effects.

Whole exome sequencing (WES) and whole genome sequencing (WGS) for genetic testing will soon be within reach of (almost) every patient. This has helped identify distinct gene variants underlying APDS and many other IEIs. Genetic diagnosis may be of considerable importance for a patient’s therapeutic management: opening the possibility for targeted therapies, e.g. pharmacological inhibition of an overactivated enzyme such as PI3Kδ (15, 16).

The aim of this review is to describe the molecular mechanism underlying APDS and update the reader on the current state of diagnosis and treatment.

## Demographics

APDS is a very rare disease with an estimated prevalence of 1-2 affected people per million. The APDS registry of the European Society for Immunodeficiencies (ESID) has registered around 200 patients from Europe and the Middle East (17, 18). The exact number of APDS patients is unknown, as many patients are not registered or not diagnosed as having an IEI. APDS can be subdivided into APDS1 and APDS2, two very similar conditions which are caused by different point mutations or deletions in either of the two genes encoding PI3Kδ subunits: *PIK3CD* and *PIK3R1* respectively (19).

## Symptoms and long-term consequences

**Figure 1:**
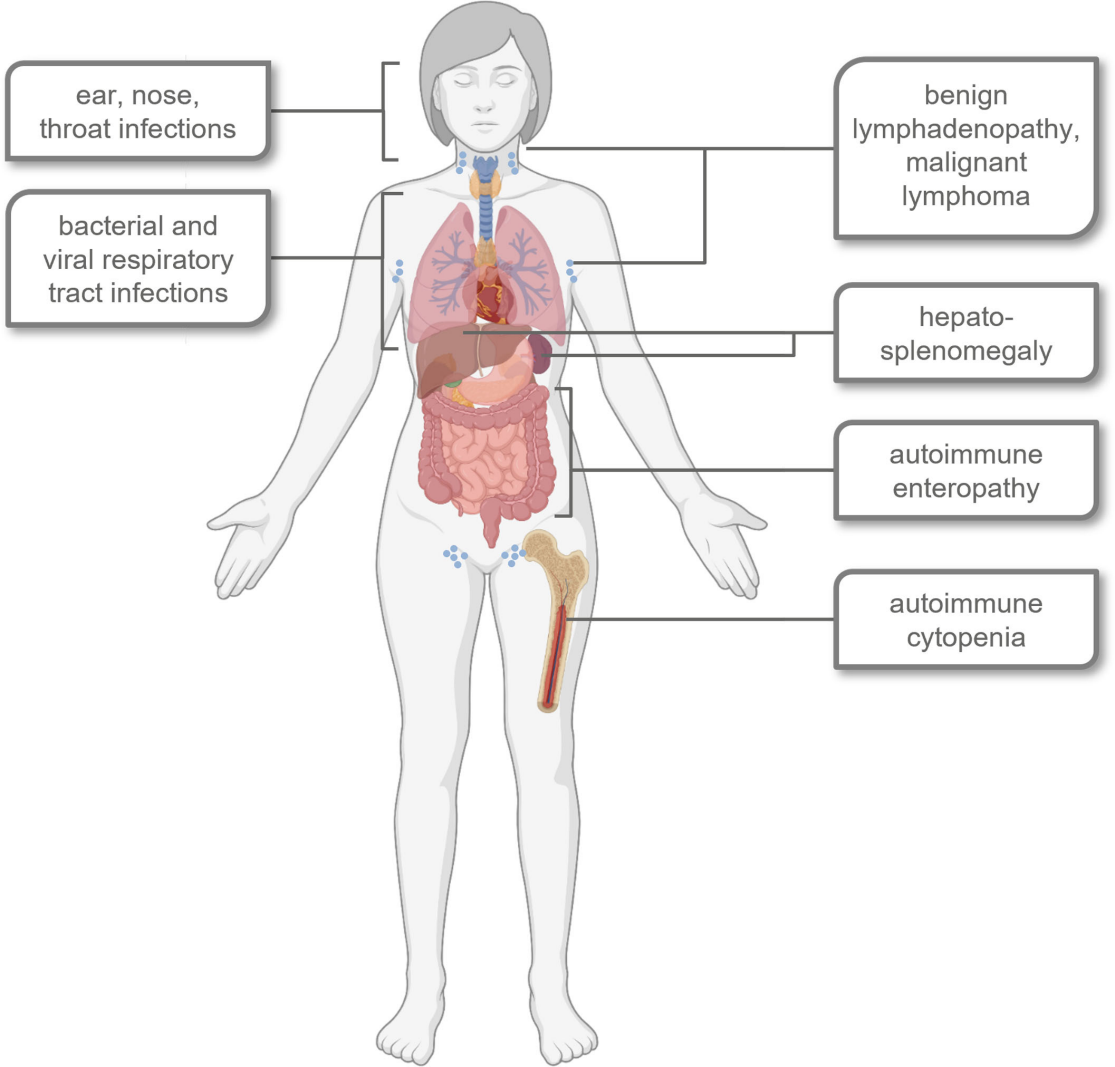
Schematic overview of typical signs, symptoms and affected organ systems in most APDS patients. Created with BioRender.com^©^.

APDS1 and APDS2 are characterized by similar symptoms, although some differences are observed, e.g. for susceptibility to certain infections or the age at onset of lymphoma (19–22). Frequent infections and autoimmunity limit patients’ quality of life while severe infections and lymphoma lower the average life expectancy of APDS patients (21, 23–25). An overview of major signs and symptoms is provided in **Figure 1**.

### Immunodeficiency

Alterations in the PI3K/Akt signaling pathway affect the function of T- and B lymphocytes. Several studies have shown a lack of naïve B cells - and a shift towards transitional B cells and plasmablasts - which can be attributed to increased rates of apoptosis as well as impaired class switch to IgG antibodies (26). In most patients, the increased susceptibility to infections manifests before the age of five years (14, 21). Since the production of plasma cells and memory B cells is also affected, patients often show hypogammaglobulinemia, a Hyper-IgM phenotype, and a selective lack of response to T cell independent vaccine stimuli (10, 20, 23, 27). Due to their inability to produce sufficient levels of neutralizing IgG and IgA antibodies, patients suffer from recurrent bacterial infections - in particular with *Streptococcus pneumoniae* (10). This often results in bronchiectases, chronic productive cough and deteriorating lung function (28).

Hyperactivation of PI3Kδ leads to a decrease in CD4+ T cells, while CD8+ T-cell levels are usually normal or increased (23), leading to an inverse CD4+/CD8+ ratio. CD8+ T cells often show signs of senescence (CD57+ expression and short telomers) and exhaustion (increased PD-1, CD160 and CD244 expression). At the same time, reservoirs of naïve CD4+ and CD8+ T cells are depleted (29, 30). Consequently, patients are often unable to eliminate viral infections, resulting in chronic infections with Herpes viruses such as Epstein-Barr-Virus and Cytomegalovirus (EBV, CMV) (29, 31). APDS is classified as a “predominantly antibody deficiency” by the IUIS (1), but since both humoral and cell-mediated immune defenses are affected, APDS could also be classified as a combined immunodeficiency.

Although APDS1 and APDS2 share several features like impaired B-cell class-switch and T follicular helper cell function, there are also differences observed in the degree of defective B cell expansion and affinity maturation, as well as intracellular signaling (32).

### Bronchiectases and disease burden

Bronchiectases primarily result from recurrent lung infections, but might also be driven by an interplay between infection and TNF-α induced p110δ expression in lung epithelium during inflammation (23). Since p110δ is constitutively active, this would also explain why APDS1 patients are more prone to developing bronchiectases compared to APDS2 patients, who carry variants in p85α. Irreversible lung damage such as bronchiectases further aggravate the problem of recurrent and chronic inflammatory lung disease. Bronchiectases significantly contribute to poor quality of life.

As APDS patients require permanent treatment and medical attention, this also creates a significant burden on the healthcare system (33). Early diagnosis and treatment may slow the development of bronchiectases and thus preserve both lung function and quality of life (25). Therefore, APDS and other IEI are an important differential diagnosis when bronchiectases occur early in life (23).

### Lymphoproliferation

Lymphoproliferation in APDS may manifest as lymphadenopathy, splenomegaly and hepatomegaly, increasing the risk for lymphomagenesis. Benign lymphoproliferation is usually the second most frequent clinical sign of APDS following repeated infections (14). Lymphadenopathy and splenomegaly in APDS patients can be massive and mimic malignant lymphoma. Besides peripheral lymph nodes mucosa-associated lymphoid tissues may be affected. Histologically, affected lymph nodes can display high rates of B-cell and T-cell proliferation (34, 35). Hemophagocytic lymphohistiocytosis (HLH)-like clinical pictures have been described, in one case followed by Hodgkin’s lymphoma. One case study reported an association of APDS2 with Kikuchi-Fujimoto syndrome (36), another presented with gut-associated T-cell lymphoproliferative disease (37, 38). Thus, careful histological examination of lymph nodes by an experienced pathologist is required to avoid unnecessary administration of cytostatic drugs (34, 35).

Recurrent adenoid hypertrophy often requires ENT surgery as patients suffer from obstructive sleep apnea. The risk for lymphoma is particularly high in APDS2 patients, with the cumulative risk estimated at 78% at the age of 40 years (21). PI3Kδ is predominantly - but not exclusively - expressed in leukocytes, which may explain why until now no predisposition for solid tumors had been observed in APDS patients (23). Long-term data in larger patient cohorts, however, seems to indicate a slightly increased risk for solid tumors as well (Maccari et al., 2023, accepted). The risk for APDS patients to develop lymphoma is aggravated by their inability to control infections with oncogenic viruses, e.g. EBV (20, 21, 30).

### Autoimmunity/autoinflammation

Overlap between immunodeficiency and autoimmunity is frequently observed in patients with various IEIs (39). Mechanisms preventing adequate defense against pathogens also affect self-tolerance and immune regulation (40, 41). Chronic activation of PI3Kδ enhances B-cell survival and increases the number of plasma cells producing autoreactive antibodies (42, 43). Studies in a PI3Kδ^E1020K^ mouse model indicate that polyclonal and self-reactive B cells expand at the expense of antigen-specific clones (26). Chronic infection with EBV promoting polyclonal B-cell proliferation may be an additional risk factor for autoimmunity (44). The predisposition for autoimmunity or autoinflammation can manifest as autoimmune cytopenias (45), chronic organ inflammation, colitis, arthritis (20, 21, 23) and granulomatous skin lesions (22, 39). Although common, frequencies of autoimmune phenomena in APDS patients vary, and may be absent (14).

### Growth retardation, syndromic features and developmental delay

Short stature and a general failure to thrive are not uncommon in APDS patients. Short stature may be a secondary effect of frequent infections and gastrointestinal inflammation. It is seen more frequently in APDS2 patients, indicating tissue-specific involvement of p110δ and p85α in somatic development and immune regulation. Indeed, other *PIK3R1* variants are associated with SHORT syndrome (short stature, hyperextensibility of joints, and/or inguinal hernia, ocular depression, Rieger anomaly, and teething delay), a condition affecting - among other things - stature and metabolism (46). In addition, neurodevelopmental delay and syndromic features are observed specifically in APDS2 patients (21, 23).

## Genetics and molecular mechanism of APDS

APDS1 is caused by heterozygous GOF variants in the *PIK3CD* gene (p110δ protein) (10, 30, 47), and APDS2 by heterozygous LOF variants in the *PIK3R1* gene (p85α protein) (11, 20). These variants likely occur sporadically but may be passed on in families for several generations or else occur *de novo* (19). A founder effect is unlikely since APDS1-affected families do not share long-range haplotypes or other markers indicating such an effect (10). APDS inheritance follows an autosomal dominant pattern (20, 30), and is characterized by high penetrance (23). Furthermore, LOF mutations of the phosphatase and tensin homolog gene (*PTEN*) cause a syndrome with similar characteristics as APDS (APDS-like) (48).

**Figure 2:**
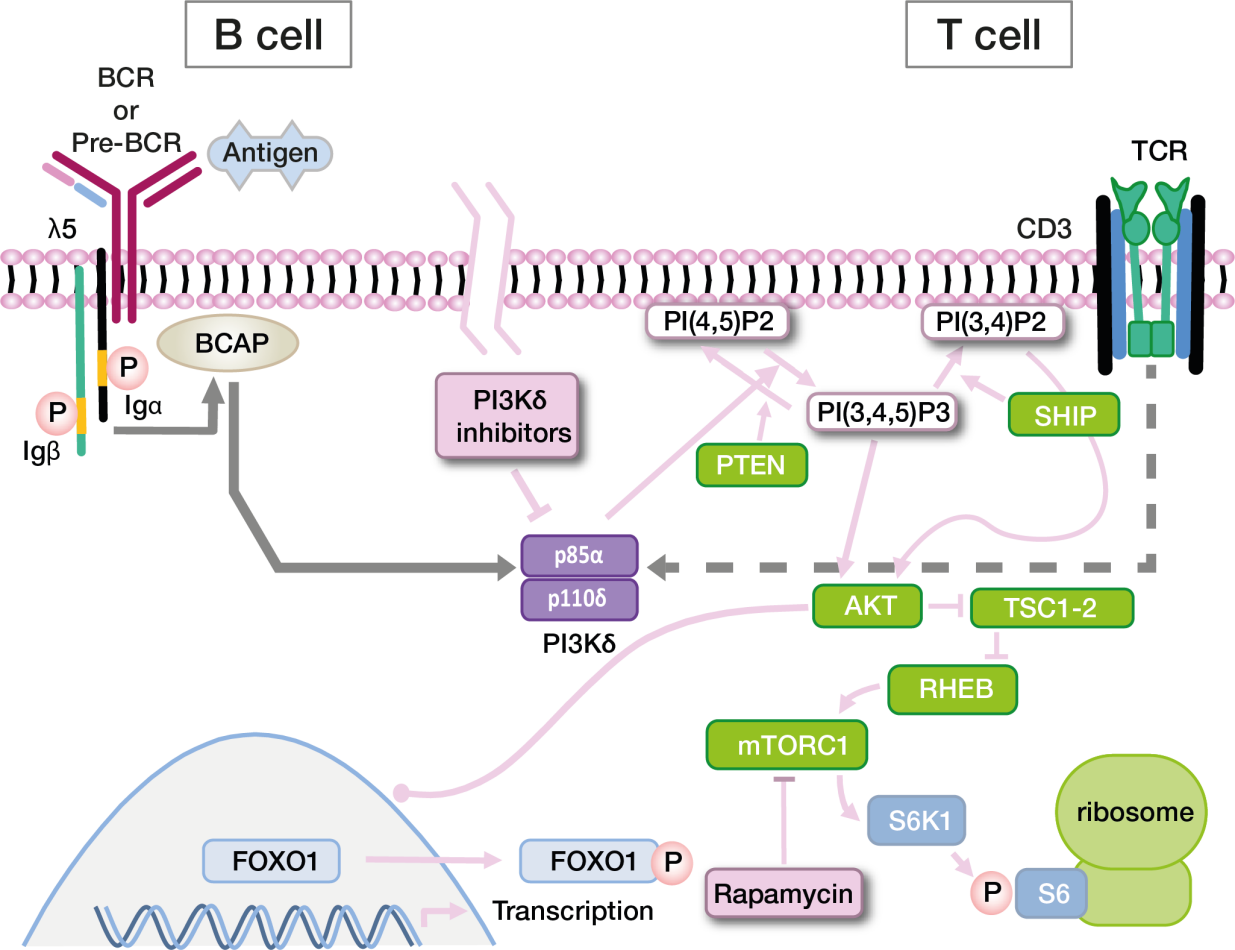
Simplified diagram of the PI3K signaling pathway in B cells and T cells, and targets for directed drug therapy. Other pathways also involving Pi3K (via IL-4R or CXCR5) are not depicted. Signaling events are extensively discussed in the text. *Abbreviations*: BCR, B cell receptor; TCR, T-cell receptor; Igα, CD79a; Igβ, CD79b; BCAP PI(*)P2, Phosphatidylinositol-*-bisphosphate; PI(*)P3, Phosphatidylinositol-*-trisphosphate; SH2, Src homology 2 domain; SHIP, SH2 domain-containing inositol-5-phosphatase; PTEN, phosphatase and tensin homologue; AKT, a SH2 containing serine/threonine kinase; TSC1-2 RHEB, RAS homologue enriched in brain; mTORC1, mTOR complex 1; S6K1, Ribosomal protein S6 kinase beta-1; FOXO1, Forkhead Box O1.

**Figure 3:**
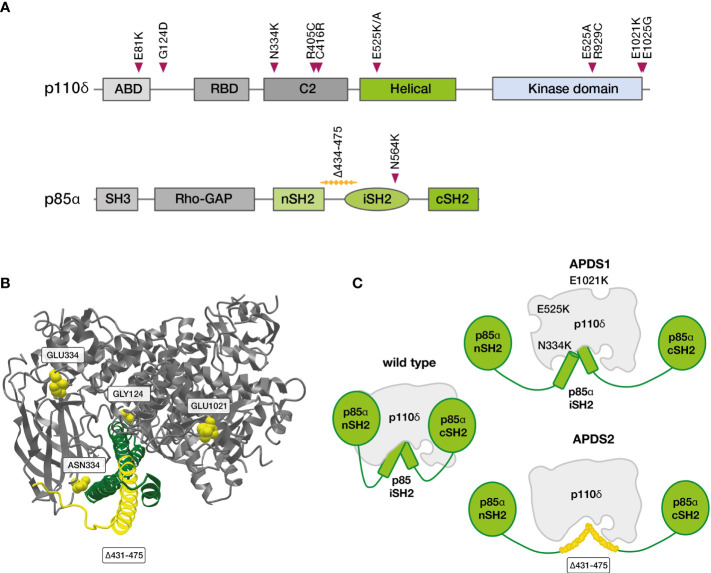
Variants in PIK3CD and PIK3R1 linked to APDS (52). **(A)** Linear depiction of p110δ and p85α proteins with confirmed, APDS-causing variants **(B)** Partial crystal structure of *Homo sapiens* p110δ (grey, positions commonly affected in APDS1 in yellow) in complex with part of the SH2 domains of *Bos taurus* p85α (green, positions commonly deleted in APDS2 in yellow), frequent APDS variants are indicated in yellow (MMDB-ID: 5DXU; image created with iCn3D webtool) (54). **(C)** Consequences of gene variants in p110δ (APDS1) and p85α (APDS2) on the PI3Kδ complex. iSH2, inter SH2 domain; cSH2, C-terminal SH2; nSH2, N-terminal SH2 domain; SH2, Src homology 2 domain.

PI3Ks are a family of lipid kinases with important roles in all eukaryotic cells. They synthesize phosphatidylinositol 3,4,5-trisphosphate (PIP3) from phosphatidylinositol-4,5-bisphosphate (PIP2). PIP3 in turn activates downstream proteins like Akt, PDK1 and mTOR. While PI3Kα and -β are active in all cells of the body, PI3Kγ and -δ are mostly - though not exclusively - active in leukocytes. PI3Kδ and its two subunits are highly relevant for the function of the adaptive immune system (49–52). p110δ is catalytically active, while p85α serves as a regulatory subunit (53). Together, these core components of the PI3K/Akt signaling pathway regulate the growth and differentiation of lymphocytes in concert with other molecules (**Figure 2**). In B cells, p110δ can be activated *via* the B-cell receptor or *via* cytokine signals. In T cells, the main activator for p110δ is T-cell receptor signaling. p85α interacts with p110δ in its resting state, inhibiting catalytic activity. Most PI3Kδ-activating variants in *PIK3CD* affect the C-terminal part of its gene product, primarily amino acid E1021 (**Figures 3A, B**) (10, 52). These variants increase baseline activity by either impairing inhibitory interactions with p85α, or increasing the affinity of p110δ for the plasma membrane, or both (55). Interaction between the nSH2 and iSH2 (SH2 = Src-homology 2) domains of p85α and between the core domain, helical domain and catalytic domain of p110δ is necessary for the inhibition of p110δ in its resting state (**Figure 3C**). Moreover, the p85α iSH2 domain is needed for establishing a solid interaction with the N-terminal p110δ adaptor binding domain (ABD) (49, 55, 56). Virtually all APDS2 patients carry a variant in the splice donor site for intron 10 of *PIK3R1*, leading to deletion of exon 10 (aa434–475) (11, 52) and to LOF in p85α (**Figures 3A, C**). As a dedicated regulator of p110δ, such LOF effectively results in GOF of the PI3Kδ complex which is the unifying characteristic of all APDS-associated gene variants. Thus, APDS elegantly illustrates how GOF in effector enzymes and LOF in their regulators have similar effects. Accordingly, LOF in regulators with primary inhibitory effects are considered GOF mutations by some authors (13).

## Diagnostic algorithm in APDS

**Figure 4:**
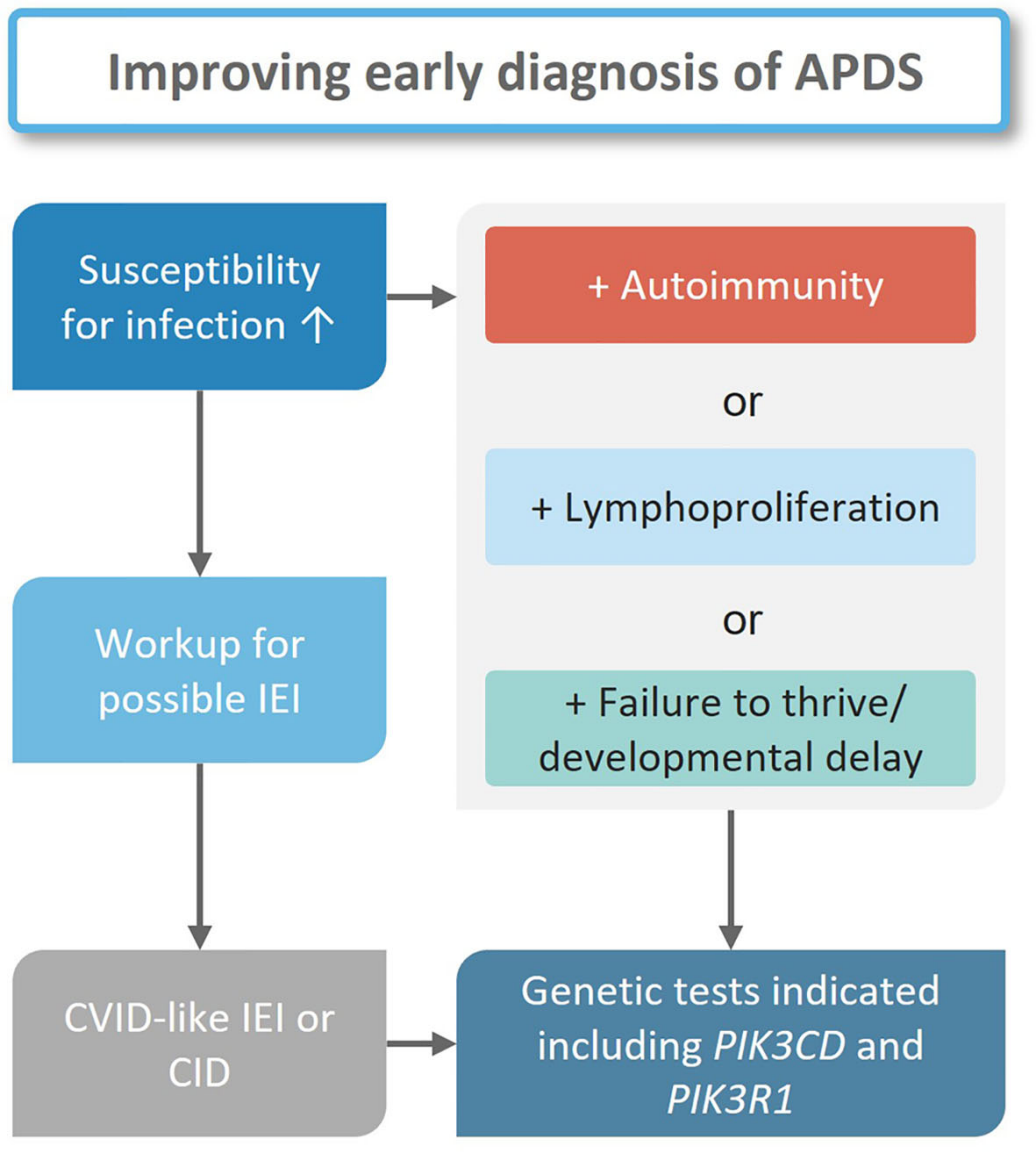
Diagnostic workflow for suspected APDS patients. In all cases with increased susceptibility to infections an appropriate workup for a possible IEI is indicated. If a diagnosis is established genetic tests should follow. If one or more other clinical findings are present in addition to infections, genetic tests should follow independent from an IEI diagnosis.

Early diagnosis of IEI, including APDS, is essential to define suitable treatments that may delay or prevent irreversible organ damage. Unfortunately, diagnosis in IEI patients is often delayed. For example, analyses of the ESID and United Kingdom Primary Immunodeficiency (UKPID) registries suggested minimum prevalences of 8.8/100000 for the Island of Ireland in 2022, and 4.4/100000 in France in 2017 (57, 58), which are well below the estimated ratio of 1/2000 in the general population. A total of 3100 IEI patients were recorded in the German immunodeficiency register PID-NET in 2019, and 4700 in 2021 (59, 60). In Germany, APDS and other IEIs are most likely underdiagnosed, however, the exact prevalence is unknown. Additionally, some patients may have been diagnosed but have not been reported to the registry (61). As a first step in IEI diagnosis, the treating pediatrician or general practitioner must appraise an unusual pattern of symptoms in their patient (**Figure 4**). Although so-called “warning signs” of IEI (62) may help identify immunologic disorders, awareness of IEI among doctors may be a limiting factor (63, 64). Such typical warning signs were proposed by the North American Jeffrey Modell Foundation (https://info4pi.org), some of which have been integrated into diagnostic guidelines. Unfortunately, these do not include signs like autoimmunity or lymphoproliferation. Expertise in diagnosing IEIs is mostly confined to physicians specialized in managing primary immunodeficiencies, and diagnosing immune-related disorders may take up to ten years, as is the case with APDS (12, 14, 65, 66), the discovery of the causative gene variants underlying APDS has helped shorten this delay. Given the sparsity and geographical distribution of clinical immunologists, there is a high probability of patients not being directly referred to a specialist. In Germany, only 220 clinical immunologists practice compared to 16.100 pediatricians, 1900 hemato-oncologists, and 900 rheumatologists (67). The General Medical Council of the United Kingdom currently (early 2023) lists 182 immunologists, as opposed to 1954 hemato-oncologists and 1426 rheumatologists (68). Patients with signs of lymphoproliferation may see an oncologist, a gastroenterologist when either enteropathy or failure to thrive are the leading symptoms, or else a rheumatologist when autoimmunity prevails. Oftentimes, patients see multiple subspecialists, and the underlying primary immunodeficiency remains unrecognized for long periods of time.

Centers specialized in IEI will ask individuals with suspected IEI for a detailed history including a family tree. Patients will then undergo a comprehensive physical examination before laboratory assays are initiated. The results of this basic assessment may help decide how likely an IEI is, and whether in-depth analyses are necessary to determine the exact nature of a suspected IEI (**Figure 4**) (64, 69–71). Phenotypical overlaps are common in IEI as is the case for APDS, which may fit under the umbrella entity of common variable immunodeficiency (CVID), although APDS is probably better classified as a combined immunodeficiency (CID). Only in exceptional cases would the clinical presentation (symptoms and lymphocyte phenotyping) suggest APDS to the experienced immunologist prior to genetic testing.

Current guidelines recommend performing advanced immunological diagnostics of suspected IEI patients prior to molecular testing (69, 70). Genetic sequencing is the accepted confirmatory step for most suspected IEIs. The treating physician may choose between gene panels, where predefined sets of genes are sequenced, or whole exome sequencing (WES). Additionally, whole genome sequencing (WGS) will complement - or even supersede - both panel and exome approaches. Given the heterogeneous clinical presentation of APDS patients, there is a potential risk of selecting the “wrong” gene panel: at worst missing the mutated gene. WES and WGS have become more affordable and are now being covered by some health insurances in developed countries, meaning many specialists may directly choose WES - or, a few, WGS - in search of a monogenic cause of the suspected IEI. It should be noted however, that only a limited number of genes from WES/WGS may be analyzed by geneticists (referred to as panel-in-exome or virtual panel). It is therefore of great value that the treating physician and the geneticist directly communicate before the genetic analysis is finalized to allow for re-evaluation of the sequencing data, and targeted filtering according to the human phenotype ontology terms (e.g. when no pathogenic variant has been identified in a given panel to explain the phenotype) (72). If a specific gene variant is identified, targeted Sanger sequencing may help segregate the variant within the family, possibly identifying other carriers or mildly affected family members.

## Identification of gene variants and variant databases

Thanks to the availability and affordability of gene and genome sequencing, an increasing number of IEIs are being mapped to the underlying genetic causes. The first variant known to cause APDS1, the E1021K variant in p110δ, was first published in 2006 (73), but APDS1 was not described as a distinct disease entity until 2013 (10). Soon thereafter, the aa434–475 deletion in p85α was recognized to cause a similar condition (11). Extensive databases on disease-causing variants, including those associated with IEI are maintained on platforms such as OMIM (www.omim.org), OrphaNET (www.orpha.net), ClinVar (www.ncbi.nlm.nih.gov/clinvar), and DisGeNET (disgenet.org) (74). However, identifying a disease-causing gene variant in the depths of the human genome remains challenging for many patients. For the identification of disease-causing variants, experts cross-check variant databases for neutral variants and use pathogenic variant prediction algorithms (75). Besides the few variants in the *PIK3CD* and *PIK3R1* genes functionally proven to be pathogenic (52), there are hundreds of variants of unknown significance (VUS), whose causality for an immunodeficiency has not yet been determined. Patients with an APDS-like presentation and abnormal PI3K/Akt signalling but without a pathogenic variant in the two APDS genes (e.g. PTEN deficiency) require further investigations. Databases like Franklin (https://franklin.genoox.com) and varsome (https://varsome.com) collect information on the pathogenicity of variants. The online tool LitVar (www.ncbi.nlm.nih.gov/CBBresearch/Lu/Demo/LitVar) offers assistance with literature research for previously reported variants. However, geneticists should not solely rely on published information as the field is constantly evolving and new information is being added to the various databases. In APDS - as is the case generally for rare hereditary diseases - the exchange between experts and the use of common platforms will greatly facilitate linking gene variants to patient phenotypes, and furthermore enrich the currently known spectrum of IEI (76). Genetic tests have the potential of making diagnostics for monogenetic diseases accessible to patients who do not yet benefit fully from modern medicine, for example due to the economic situation in their home country.

## Functional testing for PI3K activity

A number of variants have been functionally ascertained to underlie APDS, based on the correlation between genetics and symptomatology of patients (10, 11). However, to confirm a direct link between genotype and clinical phenotype, a functional proof of pathogenicity in the affected molecular pathomechanism is crucial. The primary characteristic of APDS-causing variants lies in increased activity of PI3Kδ and downstream proteins, including Akt and mTOR. Confirming a suspected diagnosis of APDS requires matching of the clinical phenotype and a (likely) pathogenic variant. This would leave patients who do not have one of the confirmed APDS variants without a diagnosis, regardless of their phenotype. Therefore in some academic settings, the overactivation of the PI3K/Akt pathway may be investigated in order to characterize the biology of APDS patient cells. This includes analysis of phosphorylation levels of downstream proteins like Akt, FOXO1, and of the ribosomal protein S6 (11, 20, 30). However, these functional tests have not found their way into clinical practice yet, which would require fresh patient samples to be shipped to a designated diagnostic center (30). The phospho-S6 ribosomal protein, however, has been proposed as a predictive marker in sarcoma (77), so it is conceivable that a phospho-S6 assay could be standardized as one possible confirmatory test for APDS. Developing functional testing for VUS in APDS could serve as a proof-of principle for IEIs characterized by GOF or LOF variants in other genes. In the future, functional testing could help assess the efficacy of PI3Kδ inhibitors in APDS and other diseases with abnormal PI3K/Akt signaling, e.g. PTEN deficiency, which could help provide existing therapy options for more patients and to monitor therapy.

## Treatment options and unmet need in APDS

### APDS in the context of other IEI

Many monogenic immunodeficiencies such as APDS are rare diseases. The disease burden of APDS patients varies greatly: from family members incidentally diagnosed with relatively mild symptoms, to patients with life-threatening or chronic airway infections, severe colitis or early-onset lymphoma (14, 24). Although it affects humoral and cellular immunity, APDS may be misclassified as CVID. Like in CVID, APDS patients suffer from frequent upper and lower airway disease, autoimmunity, lymphoproliferation and a B-cell deficiency (78). The increased risks of sepsis and lymphoma result in an inferior life expectancy (21, 23). Unlike in CVID, disease symptoms usually manifest early in childhood. Early symptom-onset is a common feature of many other monoallelic combined immunodeficiencies with similarities to APDS like CTLA-4 deficiency, NFKB1 deficiency, autoimmune lymphoproliferative syndrome variants (ALPS) or STAT3 gain-of-function (27, 28, 79–81). In terms of phenotypic severity, APDS presents as a spectrum somewhere between CVID and CID, with additional immune deregulatory features.

### Symptomatic treatment

APDS is an IEI of moderate-to-high severity with great heterogeneity in the patient population, which is reflected in the various strategies employed in managing the disease. PI3Kδ hyperactivity has multiple effects on the immune functions which makes it difficult to treat patients in a symptom-oriented fashion. Recurrent infections can be managed with immunoglobulin replacement therapy (IRT) and antibiotic prophylaxis. Indeed, most patients (70-80%, see **Table 1**) receive both IRT and antibiotics regularly or against acute infection, with some benefit observed in decreasing the frequency of respiratory tract infections (RTI) (21, 23, 24, 52). However, these options are inadequate in addressing virus reactivation or chronic viral infections, as well as in preventing lung damage. Two APDS1 and APDS2 patient cohorts documented by Coulter et al. and Elkaim et al. are reported with chronic viral infection and with progressive bronchiectases despite IRT (21, 23).

**Table 1:** Reported treatment choices for APDS patients. It should be noted that some individuals are included in more than one publication.

APDS type	Coulter et al. (2017) (23)	Elkaim et al. (2016) (21)	Maccari et al. (2018) (24)			Tessarin et al. (2020) (82)	Jamee et al. (2020) (52)	Wang et al. (2021) (83)
	APDS1 (n=53)	APDS2 (n=36)	APDS1/2 (n=68)	APDS1 (n=45)	APDS2 (n=23)	APDS1 (n=8)	APDS1/2 (n=240)	APDS1 (n=7)
Antibiotics	33	22	54			1		
IgG	46	32	44	28	16	6	151	
Immunosuppr.	16					3		
Rapamycin	6	6	27			2		3
Rituximab	8	3	8					
Steroids		5	31			4		
HSCT	5	1	8	7	1	1	31	
Splenectomy		3	5	4	1		10	

APDS, Activated PI3Kδ syndrome; HSCT, hematopoietic stem cell transplantation; IgG, Immunoglobulin G.

### Immunosuppression

To address autoimmunity and lymphoproliferation, immunosuppressants are frequently used in APDS patients, including corticosteroids and the mTOR inhibitor rapamycin (see **Table 1**). The targeted inhibitory effect of rapamycin on mTOR, which is located downstream of PI3Kδ in the PI3K/Akt pathway, provides a rationale for its use in APDS. While rapamycin appears to be particularly effective in the treatment of lymphoproliferation, its efficacy with respect to inflammation and autoimmune hemolysis is limited. It has been reported that natural antibody production may improve under rapamycin (24, 84). The drug is tolerated by most patients, but treatment interruptions or permanent discontinuation due to side effects have been documented (23, 24). The B-cell-depleting antibody rituximab has been used in APDS patients with good efficacy against hemolysis and lymphoproliferation, but invariably entails profound and long-lasting hypogammaglobulinemia. Some APDS patients (<10%) were splenectomized for autoimmune cytopenia or for diagnostic purposes (21, 23, 52).

### Stem cell transplantation

As treatment with approved drugs is often insufficient to control the debilitating and life-threatening symptoms in APDS patients, HSCT is considered a viable option with curative potential. According to the literature across all cohorts analyzed, between 10-20% of APDS patients have been treated with HSCT (see **Table 1**). HSCT is mostly offered to APDS1 patients (up to 20%) compared to APDS2 patients (<5%) (21, 23, 24, 52, 82). Life-threatening lymphoproliferation and recurrent infections despite high-dose IRT were most frequently cited as indications for HSCT (85). Conforming to data on patients with other genetically defined CID, mortality after HSCT – usually from infection – was 10-20% (85, 86). Frequent complications were graft-versus-host-disease (GvHD) and low or declining donor-type chimerism. A recent study reported a rate of 14% of patients who required a second transplantation, with one patient requiring a third (87). While HSCT has the potential to lower the frequency of infections or severity of lymphoproliferation (85, 86), manifestations in non-hematopoietic organs are likely to persist. To date, the outcome of HSCT is superior in younger patients as reported in a number of other IEIs (88). However, since the course of disease is unpredictable in young children there is a potential risk of subjecting patients with a possibly mild course of disease to potential risks of HSCT. In the literature, HSCT-related events represent the second most common cause of death in APDS patients (25).

### Targeted therapies

As discussed above, immunomodulation with the mTOR-targeting molecule rapamycin is more effective and has fewer side effects compared to the long-term use of broad-acting immunosuppressants such as corticosteroids or azathioprine. Nonetheless, rapamycin is associated with a number of side effects including diabetes-like symptoms and lung toxicity. This is because its target - mTOR - is not only involved in BCR and TCR signaling *via* PI3Kδ, but also the insulin and growth hormone signaling pathways (89). In contrast, specific inhibitors of PI3Kδ directly target the protein whose hyperactivity is at the root of APDS. Idelalisib was the first PI3Kδ inhibitor allowing for PI3Kδ activity to be regulated to normal levels in *in vitro* in samples from leukemia and lymphoma patients, but had considerable side effects *in vivo* (31, 55). Three other PI3Kδ inhibitors, nemiralisib, seletalisib and leniolisib are being tested in APDS patients. Nemiralisib showed favourable results in an animal model, decreasing mortality in *S. pneumoniae* infected mice with p110δ^E1020K^ (90). However, a study on 5 human patients treated with inhaled nemiralisib over 3 months showed good tolerance, but no measurable anti-inflammatory effects (NCT02593539) (91). Seletalisib was tested in an open label phase Ib study and demonstrated encouraging results modulating disease activity, but serious adverse events occurred in three of seven patients. The safety profile was still considered acceptable for this group of patients (92). Leniolisib, an oral inhibitor of PI3Kδ, showed promising results in an open-label trial with six APDS1 patients (15), later confirmed in a randomized, placebo-controlled phase III trial (NCT02435173). The drug was well tolerated by all patients and improved symptoms of lymphadenopathy, immune cell derangement and cytopenias. Moreover, it achieved reduction in lymph node and spleen volumes, normalized mean IgM levels, increased the number of naïve B cells and decreased the rate of transitional B cells (93). The observational period was too short for an assessment of infection load, lung function or risk of lymphoma. Currently, an extension study for leniolisib is underway (NCT02859727), in which its long-term safety and effect on patient health and quality of life are being assessed. Preliminary results confirm the favorable safety profile and long-term reconstitution of immune function, including a significant reduction of infections, shown by discontinuation of IRT in a number of probands (94). Thus, leniolisib has the potential to become a standard treatment for APDS patients from 12 years and older. Targeted therapies have the potential to normalize PI3K pathway activity in all somatic cells. In order to have a positive influence on the physical development of APDS patients, immune-modulating therapies must be initiated as early as possible. A study with leniolisib in patients aged 4-11 is recruiting patients (NCT05438407), and another with patients aged 1-6 (NCT05693129) is in preparation. Further evaluation of the long-term efficacy and safety of leniolisib is needed to assess its capability to modify future management of APDS, such as potential use as a bridging therapy prior to HSCT. Clear prerequisites for the indication of leniolisib, e.g. the genotype-phenotype correlation allowing prognosis of the expected course, remain to be determined.

## Summary and outlook

The present review has addressed the clinical, immunological, and molecular characteristics of APDS, and discussed the current challenges in the diagnosis and treatment of this complex disease. Based on our review of literature, we have identified the following key challenges in the diagnosis and treatment of APDS:

1. Underdiagnosis of APDS as a result of the heterogeneous clinical picture and lack of awareness for IEI warning signs among health care providers.
2. Limited availability and lack of standardized functional testing for APDS to identify patients where genetics remain inconclusive, e.g. variants of unknown significance
3. Limitation of conventional drug therapies to control APDS symptoms
4. Lack of consensus for predicting a severe disease course to guide decisions in personalized management including HSCT
5. Need for therapeutic options that address immunological and neurological symptoms without significant side effects

There is a need for continuing education across specialties caring for IEI patients. Multidisciplinary cooperation is essential in order to a) overcome current underdiagnosis of IEI and b) spread awareness that early and precise diagnosis usually has a direct impact on treatment decisions, including targeted therapies. In light of an increasing number of molecules for targeted treatment of specific IEI, defining the genotype of IEI should be part of diagnostic routine. Still, patients may present with typical signs of APDS but have inconclusive genetics. To characterize so-called VUS, and to identify more variants that result in PI3K pathway hyperactivity, functional testing procedures within reach of specialty clinics need to be validated. Traditional management of APDS is based on antibiotic or antiviral treatment and prophylaxis for recurrent infections, IRT as well as immunosuppressive agents to control symptoms of immune dysregulation (21, 23, 52). Since the current therapeutic options are sometimes insufficient to adequately control symptoms, HSCT as a potentially curative treatment could be an alternative for selected patients. Especially in younger patients at risk for severe course of disease, HSCT can improve immune function, and reduce frequency of infections and severity of lymphoproliferation (85, 86). However, markers predicting severity of disease and individual risk to develop lymphoma are currently missing, so risk *vs*. benefit assessment remains a true challenge. Large observational studies such as the ESID APDS registry are best suited to identify such markers. The promising results for targeted treatment with leniolisib suggest that targeted therapy could become a viable option in APDS and other monogenetic IEI. Leniolisib is a convincing example of a targeted disease-modifying drug developed for the treatment of a distinct IEI. The drug was approved by the FDA (95) and is awaiting approval by the EMA.

Since both HSCT and targeted therapies have advantages and shortcomings, both approaches need to be discussed depending on a patient’s condition and disease severity. Better understanding of the pathomechanisms underlying other IEIs holds the prospect of making novel or repurposed targeted therapeutics available for patients, either newly diagnosed or with progressive disease. The relative simplicity of their application could also make them a reasonable option for patients living in countries where facilities for stem cell transplantation are not yet available. Targeted treatments may prevent or delay disease progression and contribute to a better quality of life in individuals with IEI.

## Author contributions

SV is the first author and was the main person responsible for the literature search and writing of the manuscript text. Furthermore, he was responsible for drafting the outline. VW supported the first author in the conceptualization of the article, contributed content ideas, checked the content for scientific correctness, and assisted in the structuring of the article. CS contributed her expert clinical and scientific knowledge to the content of the article, provided additional literature sources, and helped harmonize the structure of the content. Furthermore, she provided critical feedback on the messaging of the article regarding methods of diagnosis and treatment options for APDS.
